# A Multicenter, Randomized, Double-Blinded, Placebo-Controlled Clinical Trial to Evaluate the Efficacy and Safety of a Krill Oil, Astaxanthin, and Oral Hyaluronic Acid Complex on Joint Health in People with Mild Osteoarthritis

**DOI:** 10.3390/nu15173769

**Published:** 2023-08-29

**Authors:** W. Stephen Hill, Margaret H. Dohnalek, Yejin Ha, Seok-Jung Kim, Jae-Chul Jung, Seung-Baik Kang

**Affiliations:** 1 US Nutraceuticals, Inc. d/b/a Valensa International, Eustis, FL 32726, USA; s.hill@valensa.com (W.S.H.); m.dohnalek@valensa.com (M.H.D.); 2 NOVAREX Co., Ltd., 80, Osongsaengmyeong 14-ro, Osong-eup, Chueongju-si 28220, Republic of Korea; yj9113@novarex.co.kr; 3 Department of Orthopedic Surgery, Uijeongbu St. Mary’s Hospital, College of Medicine, The Catholic University of Korea, Cheonbo-ro, Uijeongbu-si 11765, Republic of Korea; peter@catholic.ac.kr; 4 Department of Orthopedic Surgery, Seoul National University College of Medicine, Boramae Hospital, Seoul 07061, Republic of Korea

**Keywords:** FlexPro MD^®^, osteoarthritis, inflammation, joint pain, krill oil, astaxanthin, hyaluronic acid

## Abstract

Osteoarthritis is a significant global health problem. Many patients seek more effective alternatives to nonsteroidal anti-inflammatory medicines or commercial supplements to manage joint pain and inflammation. FlexPro MD^®^ (FP-MD) combines krill oil, astaxanthin, and lower molecular weight hyaluronic acid to support joint health. A 12-week, randomized, double-blind, placebo-controlled trial compared the efficacy and safety of FP-MD and placebo once daily in participants (n = 100) with mild osteoarthritis of the knee or hip joint. For the primary endpoint of joint pain score, per-protocol participants (n = 75) in the FP-MD group (n = 37) had a statistically significantly greater mean reduction from baseline in the Korean Visual Analog Scale (K-VAS) at week 12 compared with participants in the placebo group (n = 38) (20.8 ± 16.16 mm vs. 10.6 ± 17.58, *p* = 0.0105). The Korean Western Ontario and McMaster Universities Osteoarthritis Index (K-WOMAC) total score was also significantly improved in the FP-MD group at week 12 compared with placebo (−13.0 ± 13.62 vs. −5.5 ± 18.08, *p* = 0.0489), especially an improvement in pain score (−2.5 ± 2.92 vs. −1.3 ± 3.94, *p* = 0.02635). FP-MD group had greater improvement in joint function scoring by investigator assessment (*p* = 0.0127) and by group participants (*p* = 0.0070). A statistically significantly greater number of patients reported adverse events in the placebo group compared with the FP-MD group (16% vs. 4%, *p* = 0.0455), most commonly gastrointestinal disorders in both of the groups. These findings suggest that FP-MD is well tolerated and can be effectively used to address joint pain in patients diagnosed with mild osteoarthritis, the main symptom of this condition.

## 1. Introduction

Arthritis is a chronic disease that affects millions of people worldwide. According to the Global Burden of Disease published in 2017, approximately 300 million people suffer from musculoskeletal disorders in 195 countries. Osteoarthritis and rheumatoid arthritis account for 19.3% and 1.3% of people affected by musculoskeletal disorders, respectively [1].

The global prevalence of osteoarthritis has increased by 48% from 1990 to 2019, with the highest prevalence in North America, specifically in people 50 to 60 years of age [2]. The pathogenesis of osteoarthritis is not uniform, and various causes have been reported. These include general factors such as age, sex, genetic reasons, and obesity, as well as external factors such as muscle weakness, leg length imbalance, and joint damage [3].

There is no cure for osteoarthritis, and most patients use a variety of treatment methods to relieve symptoms such as joint pain and stiffness. Medication treatments include oral acetaminophen, corticosteroids, or nonsteroidal anti-inflammatory drugs (NSAIDs), application of anti-inflammatory patches, or intraarticular injections of corticosteroid or hyaluronic acid (HA) to reduce inflammation and increase joint flexibility, respectively [4]. Physical therapies, such as diathermy, exercise therapy, and ultrasound therapy, are also used [5].

The most commonly used treatment for osteoarthritis is medication therapy, which aims to relieve pain and inflammation, improve joint function, and limit disease progression. Medication therapy should consider the patient’s underlying disease, other medications being taken, comorbidities, and patient preferences. Oral NSAIDs are the most commonly used medication to manage osteoarthritis. However, NSAID therapy can lead to various side effects, such as gastrointestinal tract disorders, cardiovascular issues, liver damage, and decreased renal function [6].

Interest in functional joint health ingredients with few side effects, such as soy isoflavones, N-acetylglucosamine, and methylsulfonylmethane (MSM), is increasing. These ingredients are known to regulate collagen degradation and synthesis, have anti-inflammatory activity, and inhibit joint damage by promoting cartilage synthesis [7,8,9]. FlexPro MD^®^ (FP-MD) is a unique formulation of functional ingredients, specifically, omega-3 fatty acids (e.g., eicosapentaenoic [EPA] and docosahexaenoic acid [DHA]), astaxanthin, and a proprietary lower molecular weight HA. It is designed to address the root cause of joint breakdown and pain caused by oxidative stress. The FP-MD formulation delivers omega-3 fatty acids (e.g., EPA, DHA) in the form of krill oil to promote optimal omega-6:omega-3 ratios; a stable, easily absorbable, lipid-soluble antioxidant in the form of astaxanthin; and low molecular weight HA (500–50,000 Da) for improving joint lubrication. Of note, the phospholipid-bound omega-3 polyunsaturated fatty acids (PUFAs) in krill oil have been shown to improve the oral absorption of HA [10]. In addition, the oral bioavailability of astaxanthin is increased in the presence of emulsifiers, such as the phospholipids found in krill oil [11,12].

The objective of this study was to evaluate the efficacy and safety of FP-MD compared with a placebo in addressing joint pain in participants diagnosed with mild degenerative osteoarthritis after 12 weeks of supplementation.

## 2. Materials and Methods

### 2.1. Test Products

FlexPro MD^®^ is a commercially available dietary supplement containing a combination of *Euphausia superba* Antarctic krill oil (321 mg, Superba^®^, Aker BioMarine Antarctic US LLC; Metuchen, NJ, USA), natural astaxanthin purified from *Haematococcus pluvialis* (2 mg, Zanthin^®^ Natural Astaxanthin), and a proprietary HA produced from fermentation by *Streptococcus zooepidemicus* (30 mg, Flexonic^®^ sodium hyaluronate (the sodium salt of HA), Valensa International; Eustis, FL, USA). FP-MD is a 600 mg soft capsule consisting of a reddish-brown, oily liquid. The placebo was an identically appearing soft capsule containing palm oil, olive oil, soybean oil, and beeswax (Table 1). Participants were instructed to take one capsule once daily for 12 weeks; no instructions were provided to take the capsule with or without food. Participants were also advised to maintain their regular diet and physical activity levels during the 12-week study period and not to consume other dietary sources of krill oil, *Haematococcus* (astaxanthin), or sodium hyaluronate. At the end of the study, the test product adherence rate was calculated as the number of capsules taken divided by the number of capsules dispensed ×100%.

### 2.2. Ethics

The study was conducted in accordance with the Declaration of Helsinki and approved by the respective Institutional Review Board (IRB) Research Ethics Review Committees of Boramae Hospital (IRB No. 30-2018-62) and Uijeongbu St. Mary’s Hospital (UIRB-New 2020092-013). All participants provided written informed consent before any screening assessment was completed. The study was retrospectively registered with the Clinical Trials Information Service (CRIS) of the Republic of Korea on 8 August 2023 (KCT0008749).

### 2.3. Participants and Eligibility Criteria

Participants for this clinical trial were recruited through online and offline advertisements for joint health. Korean men and women selected for inclusion were between the ages of 30 and 75 years with a Korean pain Visual Analog Scale (K-VAS) score of ≥30 mm [13] and Grade I or II for knee or hip joints based on the Kellgren and Lawrence scale for radiographic classification of osteoarthritis [14].

Potential participants were excluded if they had the following: (1) arthritis caused by specific factors other than degeneration as determined by the investigator; (2) joint spacing of ≤2 mm; (3) periarticular osteophyte phenomena, irregular articular surface, or subchondral bone cysts of the joints with moderate arthritis; (4) clinically significant cardiovascular, immune, infectious, and oncologic diseases; (5) concurrent treatment for gastritis or gastric ulcer; (6) uncontrolled hypertension (>160/100 mm Hg); (7) uncontrolled diabetes (fasting blood glucose > 180 mg/dL or starting a new diabetes medication within previous 3 months); (8) thyroid disease (thyroid stimulating hormone of <0.1 μU/mL or ≥10 μU/mL); (9) aspartate transaminase or alanine transaminase levels > 3 times the upper limit of normal; (10) creatinine level > 2 times the upper limit of normal; (11) pregnant or lactating women; (12) used arthritis-related medicines or dietary supplements within 2 weeks of screening; (13) mental illness (schizophrenia, depression, drug addiction, etc.); (14) received treatment for degenerative arthritis within 2 weeks of screening; (15) participated or planned to participate in other clinical trials within 2 months of baseline; (16) sensitivities or allergies to krill oil, astaxanthin, or HA; (17) been deemed inappropriate for study participation by the investigator.

### 2.4. Experimental Design

This study was a multicenter, randomized, double-blind, placebo-controlled clinical trial of 93 participants who were randomized 1:1 to receive either an FP-MD or placebo soft gel once daily by mouth for 12 weeks. The random allocation sequence was generated by a randomization program of the SAS^®^ system. In cases where participants violated the clinical trial protocol, such as failing to meet eligibility criteria, withdrawal of consent, non-adherence, or experiencing adverse events (AEs) that significantly impacted their safety or the study results, they were dropped after consultation with the investigator.

### 2.5. Primary Efficacy Assessment

The K-VAS is a highly reliable and valid tool for assessing joint pain that has been used in clinical trials [13,15]. Participants indicated their pain severity at 12 weeks using a 100 mm straight line, where 0 mm indicated no pain and 100 mm indicated unbearable pain.

### 2.6. Secondary Efficacy Assessments

#### 2.6.1. K-VAS and Korean Medical Outcome Study 36-Item Short Form (KSF-36)

A change of K-VAS at 6 weeks was validated to check improvement of pain index during the middle of the dosage period. KSF-36 is the Korean version of the SF-36, a multidimensional generic health-related quality-of-life instrument consisting of 36 items. It is a general measurement tool that can measure overall health status, not targeting a specific age, disease condition, or treatment group. The KSF-36 score is expressed as a value between 0 (representing the lowest health status) and 100 (representing the highest health status) by converting the raw data.

#### 2.6.2. Korean Western Ontario and McMaster Universities Osteoarthritis Index™ (K-WOMAC)

The WOMAC, a validated joint pain, stiffness, and physical function scale developed for patients with degenerative arthritis of the lower limb (e.g., knee and hip joints) [16], was adapted to Korean culture using a modified questionnaire. The K-WOMAC has been validated and used in various clinical trials [15,17,18]. Consistent with the WOMAC, the K-WOMAC has a total of 24 questions and consists of 3 subscales: pain (5 questions), stiffness (2 questions), and physical function (17 questions). Each question is scored from 0 to 4, with lower scores indicating less pain or stiffness and better function. The K-WOMAC was assessed at baseline and weeks 6 and 12.

#### 2.6.3. Serum C-Reactive Protein (CRP)

CRP is one of the plasma proteins that significantly increases in persons with inflammatory diseases or in the event of body tissue necrosis and is an acute phase protein. It has characteristics that cannot be observed in typical immunoglobulins, such as a rapid increase in levels within 6 to 24 h when a problem occurs in the body and a rapid decrease and disappearance within 24 h upon recovery. CRP data are very useful for determining the presence and severity of inflammatory or tissue-disrupting diseases as well as follow-up observations and determination of prognosis. It was measured at screening and week 12.

#### 2.6.4. Urinary C-Telopeptide of Type II Collagen (CTX-II)

CTX-II is one of the major biomarkers for the evaluation of osteoarthritis, and the CTX-II is measured to determine the degree of cartilage wear using blood or urine [19]. About 10 mL of urine was collected at screening and week 12, stored frozen until the end of the clinical trial, and discarded after analysis at an external analysis agency.

#### 2.6.5. Improvement Assessment

At weeks 6 and 12, participants and investigators assessed the degree of improvement compared with baseline using a 5-point improvement assessment score (Table 2). Participants completed this assessment without the investigator present and before the investigator’s assessment of improvement.

### 2.7. Safety Evaluations

Safety evaluations of test products included monitoring of AEs, conducting clinical pathology tests (e.g., hematologic, blood chemistry, blood lipid, and urine tests), measuring vital signs (e.g., pulse and blood pressure), checking physical measurements (body weight), and analyzing electrocardiogram results for abnormal findings throughout the study.

Adverse events were assessed throughout the study. Follow-up was conducted on all AEs until they disappeared, stabilized, or became definable symptoms.

### 2.8. Statistics

#### 2.8.1. Sample Size

No formal statistical power analysis was completed to determine the study sample size. The enrollment goal was 50 participants per group to account for an expected dropout rate of 25%.

#### 2.8.2. Analysis Sets

The per protocol (PP) set included all participants who completed the study and had no protocol violations. The safety set included all randomized participants who received at least one dose of test product.

Analysis of demographic and lifestyle data was based on the PP set. Efficacy analyses were based on the PP set to identify a treatment effect under optimal conditions. Safety analyses were based on the safety set.

#### 2.8.3. Baseline Characteristics Analyses

Descriptive statistics were provided for demographic and lifestyle data, and comparisons between groups were made using the 2-sample *t*-test or Wilcoxon rank–sum test depending on the normality assumption. Categorical data were presented as frequencies and percentages for each level, and a Chi-square or Fisher exact test was used to test for independence.

Efficacy data were analyzed by calculating the mean and standard deviation using appropriate descriptive statistics. All tests of statistical significance were 2-tailed with alpha < 0.05. Analysis of covariance (ANCOVA) was used for efficacy endpoints to adjust for participant baseline characteristics.

#### 2.8.4. Primary Efficacy Analysis

The change in mean K-VAS scores from baseline to week 12 was analyzed using a paired *t*-test or Wilcoxon signed–rank test according to whether normality was satisfied. To compare the changes between FP-MD and placebo groups, it was determined whether there was a statistically significant difference based on a 2-sample *t*-test or Wilcoxon rank–sum test based on ANCOVA and whether normality was satisfied.

#### 2.8.5. Secondary Efficacy Analyses

Mean changes from baseline in the VAS at week 6 and the K-WOMAC total and subscale scores at weeks 6 and 12 were analyzed using a paired *t*-test or Wilcoxon signed–rank test according to whether normality was satisfied. To compare the changes between treatment groups, it was determined whether there was a statistically significant difference based on a 2-sample *t*-test or Wilcoxon rank–sum test based on ANCOVA and whether normality was satisfied. The participant and investigator improvement assessment scores were analyzed using a 2-sample *t*-test or a Wilcoxon rank–sum test depending on whether normality was satisfied.

#### 2.8.6. Safety Analyses

The safety analysis evaluated the type and incidence of AEs, their severity, and their association with test products. Additionally, clinical pathology tests (hematologic, blood chemistry, and urine tests), vital signs (pulse, blood pressure), physical measurements (weight), and electrocardiogram test results were summarized descriptively. All AEs were coded according to the Medical Dictionary for Regulatory Activities (MedDRA).

All statistical analyses were conducted using SAS (Version 9.4, SAS^®^ Institute, Cary, NC, USA).

## 3. Results

**Figure 1:**
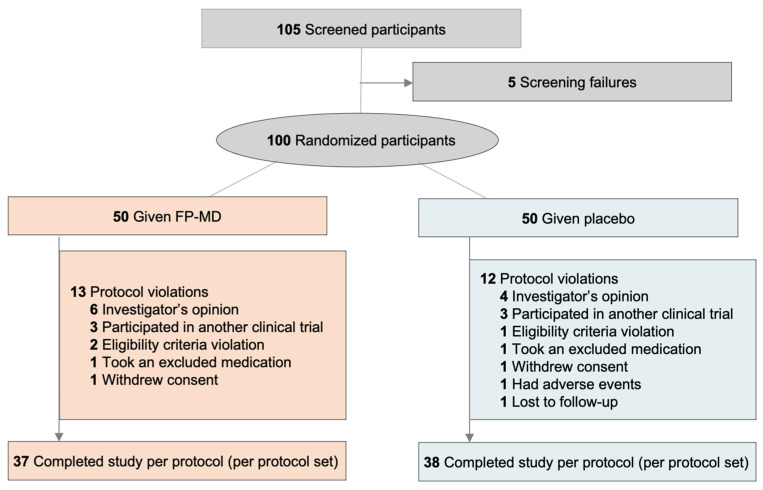
Consolidated Standards of Reporting Trial (CONSORT) participant disposition diagram for the per-protocol set. FP-MD, FlexPro MD^®^.

This study was completed between 21 December 2018, and 25 October 2019, at two clinical sites in Korea. Of 105 people screened, 5 did not meet eligibility criteria, and 100 participants were randomized to FP-MD (n = 50) or placebo (n = 50) (safety set, Figure 1). In the FP-MD group, two withdrew consent or took concomitant prohibited medications. In the placebo group, 5 participants withdrew consent, experienced AEs, were lost to follow-up, violated eligibility criteria, or took concomitant prohibited medications, resulting in a total of 93 participants completing the clinical trial (FP-MD, n = 48; placebo, n = 45). Figure 1 shows the final PP set, along with the list of protocol violations.

### 3.1. Participant Characteristics

Table 3 shows a comparison of the participant baseline demographic and lifestyle characteristics. The FP-MD group had 16 men (43.2%) and 21 women (56.8%), whereas the placebo group had 15 men (39.5%) and 23 women (60.5%) (*p* = 0.7403). The mean age of both groups was similar (*p* = 0.2370). Further, there were no statistically significant differences between groups in exercise, smoking status, amount or duration, or alcohol consumption. There were no significant differences in baseline characteristics between groups; therefore, the comparability between groups was suitable for the evaluation of differences in efficacy and safety.

### 3.2. Joint Pain K-VAS

**Figure 2:**
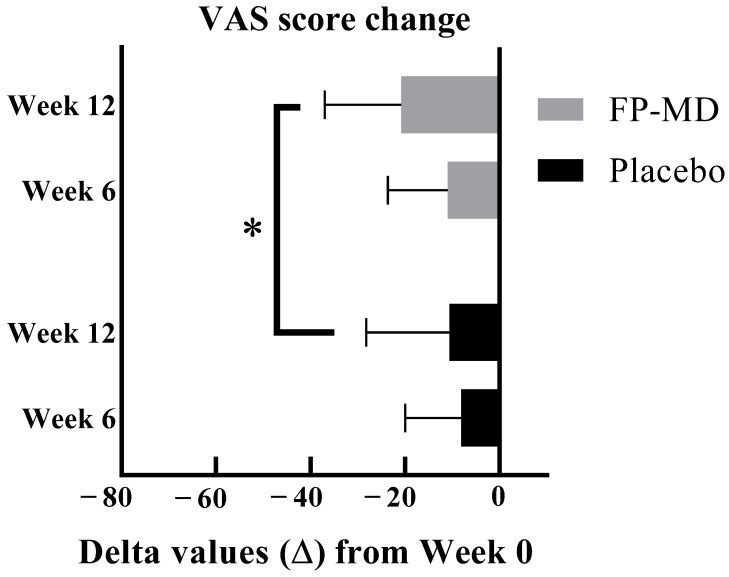
Effect of FP-MD on VAS score at weeks 6 and 12. FP-MD, FlexPro MD^®^; VAS, Visual Analog Scale. * *p*-value based on 2-sample *t*-test.

Mean joint pain K-VAS values were similar for the FP-MD and placebo groups at baseline (Table 4). For the primary efficacy endpoint (the difference between groups in the change in joint pain K-VAS scores from baseline to week 12), participants in the FP-MD group had a statistically significantly greater mean reduction in joint pain compared with participants in the placebo group (20.8 ± 16.16 mm vs. 10.6 ± 17.58, *p* = 0.0105 (unadjusted); *p* = 0.0255 (adjusted for baseline factors and adherence)) (Table 4, Figure 2).

For the secondary efficacy endpoint (the difference between groups from baseline to week 6 in the change in joint pain K-VAS scores), there was no statistically significant difference between groups (Table 4). The changes within groups from baseline to week 6 were statistically significant (FP-MD, 11.0 ± 12.62 mm (*p* < 0.0001); placebo, 8.1 ± 11.87 mm (*p* < 0.0001)).

### 3.3. K-WOMAC

**Figure 3:**
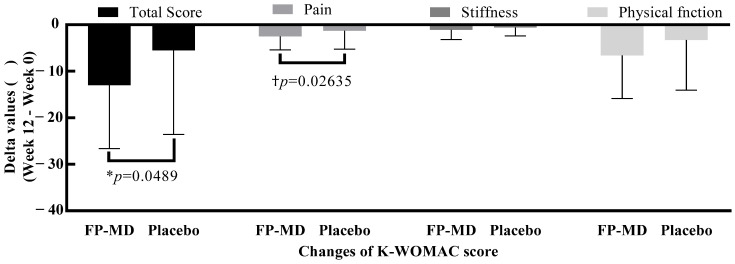
Changes in K-WOMAC score after 12 weeks of FP-MD intake compared with placebo. FP-MD, FlexPro MD^®^; K-WOMAC, Korean Western Ontario and McMaster Universities Osteoarthritis Index. * *p*-value based on 2-sample *t*-test. † *p*-value based on Wilcoxon rank–sum test.

Baseline K-WOMAC total and subscale scores were similar for FP-MD and placebo groups (Table 5). For the FP-MD group, within-group mean changes from baseline to weeks 6 and 12 were statistically significantly lower (improved) for the total K-WOMAC score and all subscale scores (*p* < 0.01 for all comparisons; Table 5). At week 12, the mean change from baseline in the total K-WOMAC score was statistically significantly lower in the FP-MD group compared with the placebo group (−13.0 ± 13.62 vs. −5.5 ± 18.08, *p* = 0.0489 (unadjusted); *p* = 0.1063 (adjusted for baseline factors and adherence)) (Figure 3).

Mean changes from baseline in the subscale scores were not statistically significantly different between FP-MD and placebo groups at week 6 (Table 5). However, at week 12, pain, stiffness, and physical function subscale scores were significantly lower in participants taking FP-MD (Table 5; Figure 3).

### 3.4. Investigator and Participant Improvement Assessment Scores

**Figure 4:**
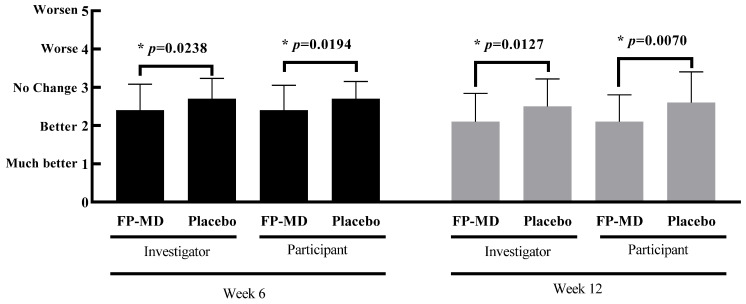
Effects of oral intake of FlexPro MD^®^ (FP-MD) on joint improvement assessment score evaluation by investigator and participants at weeks 6 and 12 compared with placebo. * *p*-value based on Wilcoxon rank–sum test.

Both investigator and participant joint improvement assessment scores were statistically significantly lower, indicating a greater degree of improvement at weeks 6 and 12 (Table 6; Figure 4).

### 3.5. Serum CRP and Urinary CTX-II Levels

Analyses of changes in serum CRP and urinary CTX-II levels showed no statistically significant differences between FP-MD and placebo groups after 12 weeks of intake. Serum CRP level after 12 weeks of intake using the PP set showed a mean decrease of 0.04 ± 0.22 mg/dL (*p* = 0.7309 vs. baseline) for the FP-MD group and a mean increase of 0.02 ± 0.11 mg/dL (*p* = 0.8148 vs. baseline) for the placebo group, but there was no statistically significant difference between groups. Urinary CTX-II levels after 12 weeks of intake using the PP set showed a mean increase of 26.03 ± 280.22 ng/mmoL (*p* = 0.3972 vs. baseline) for the FP-MD group and a mean decrease of 66.33 ± 178.84 ng/mmoL (*p* = 0.0601 vs. baseline) for the placebo group, but there was no statistically significant difference between groups.

### 3.6. Safety Evaluation

Adverse events are summarized in Table 7. A statistically significantly greater number of patients reported AEs in the placebo group compared with the FP-MD group (16% vs. 4%, *p* = 0.0455). The most common AEs were gastrointestinal disorders in both the FP-MD (2%) and placebo groups (6%). Most AEs in both groups were mild or moderate; only one AE in the placebo group was rated as severe. All AEs were categorized by investigators as thought to be unrelated or clearly unrelated to test products. One serious AE (SAE) occurred in the FP-MD group (accidental injury requiring surgery) and two in the placebo group (pregnancy, intentional overdose of sleeping medication). No SAEs were thought to be related to test products. Only one participant dropped out of the study due to AEs, and this was in the placebo group.

Hematologic tests (Table S1), blood chemistry and lipid tests (Table S2), urine tests, vital signs (pulse, blood pressure) (Table S3), physical measurements (weight) (Table S3), and electrocardiogram analyses (Table S4) showed no statistically significant differences between the FP-MD and placebo groups during the 12-week study period.

## 4. Discussion

Osteoarthritis affects people of all ages, but it is more common in older individuals, affecting nearly half of adults over the age of 65. It is reported to be more prevalent in women than men and can be caused by various factors, including obesity, low mobility, and frequent intense exercise [14]. Arthritis severity is classified according to the Kellgren and Lawrence classification system, which includes five severity criteria (Grade 0: none, Grade 1: doubtful, Grade 2: minimal, Grade 3: moderate, Grade 4: severe) [20].

Most degenerative arthritis trials focus on participants with Kellgren and Lawrence severity between Grade 2 and Grade 3 [21,22]. However, this trial confirmed the efficacy of FP-MD as a dietary supplement in addressing the symptoms associated with joint pain and joint function in participants who presented with degenerative arthritis of Grade 1 or Grade 2 severity at screening. None of the patients in the FP-MD group had a worsening of their joint pain or joint function that would be associated with a possible progression of their degenerative arthritis. Compared with the placebo group, joint pain K-VAS scores and K-WOMAC total and subscale scores were significantly improved at week 12. These findings suggest that FP-MD intake for 12 weeks reduced joint pain and stiffness and improved physical function. In addition, statistically significant differences between FP-MD and placebo groups in the improvement assessment scores at both time points suggest investigators and participants recognized the effectiveness of taking FP-MD in alleviating arthritis symptoms as early as week 6.

Clinical research on natural product-based medicines and natural product ingredients for improving osteoarthritis is actively underway. Studies are being conducted on natural medicines that directly target specific mechanisms, such as arthritis inflammatory mechanisms (e.g., interleukin [IL-1], tumor necrosis factor [TNF]-α), cartilage mechanisms (e.g., Wnt signaling pathway, cathepsin-K, MMP/ADAMTS, growth hormone inhibitors), subchondral bone destruction, and pain reduction [23]. Furthermore, research on safe natural product medicines or dietary supplement ingredients using natural products is actively being carried out. Studies on joint pain indicators such as WOMAC and VAS have reported positive results after ingestion of functional ingredients such as Flavocoxid, *Curcuma domestica*, purple passion fruit peel, chicory root, *Boswellia serrata*, and *Zingiber officinale* as dietary supplements for up to 12 weeks [24].

Most studies have focused on evaluating the efficacy of a single substance, as previously noted, and there are few clinical trials confirming the synergistic effect of multiple compounds. Except for clinical trials confirming the synergistic effects of a blend of chondroitin, glucosamine, and MSM—representative natural supplements for degenerative arthritis—no clinical trials of more than three compounds have been identified. This highlights the need for further research on the synergistic effects of multiple compounds [25,26].

The main components of FP-MD are krill oil, astaxanthin, and HA, which have shown beneficial effects on joint health in both preclinical [27,28] and clinical studies [29]. Synergistic effects of these three ingredients have been shown in animal models induced by lipopolysaccharide (LPS) and monosodium iodoacetate (MIA). Krill oil, extracted from *Euphausia superba* in the Antarctic Ocean, contains phospholipid-bound omega-3 fatty acids, including EPA and DHA. Multiple preclinical studies have demonstrated the anti-inflammatory effects of omega-3 fatty acids and a reduction in joint pain [30,31]. A recent clinical trial of krill oil demonstrated significant improvements in knee pain, stiffness, and physical function as measured by the WOMAC in adults with mild to moderate knee osteoarthritis [32]. The Superba^®^ krill oil used in FP-MD has undergone various studies to evaluate the extraction process, ingredient properties, and in vivo toxicity, confirming its safety and purity over many years [33,34,35].

Astaxanthin is a natural keto-carotenoid that exhibits molecular target activity in various diseases, including antioxidant, anticancer, antidiabetic, and protective effects in cardiovascular and neurologic diseases, as well as immunostimulating effects and is mainly purified from *Haematococcus pluvialis* and *Phaffia rhodozyma* [36]. The astaxanthin used in FP-MD was purified from *Haematococcus pluvialis* and has been shown in preclinical studies to reduce cartilage damage, prevent the progression of osteoarthritis, and have antioxidant effects [37,38]. *Haematococcus pluvialis*, a common source of astaxanthin, are freshwater microalgae that contain the highest natural concentration of natural astaxanthin [39]. An analysis of 328 reports of reported efficacy activity of astaxanthin supplements, particularly derived from Haematococcus, confirmed the greatest efficacy in sore muscles and joints (146 studies), back pain (48 studies), and osteoarthritis (20 studies), with 95% of osteoarthritis studies (19 of 20) reporting reduced pain and improved function [40].

Hyaluronic acid, isolated by Karl Meyer and John Palmer in 1934 [41], now plays a significant clinical role in several medical fields, including ophthalmology, joint pathology, skin repair, skin remodeling, vascular prosthesis, adipose tissue engineering, nerve reconstruction, and cancer treatment [42]. Hyaluronic acid, the most commonly prescribed natural ingredient for joint injections, has both lubricating and shock-absorbing properties in joints. Supplementation with injected HA fillers or oral administration of HA improves joint function by reducing inflammation in preclinical studies [43]. Clinical studies have also reported that intra-articular injections can improve joint mobility and reduce pain in patients with knee osteoarthritis [44]. Oral administration of HA has also been shown to improve clinical symptoms, including pain reduction, in clinical studies of patients with knee osteoarthritis [45,46,47]. The method of producing HA through *Streptococcus zooepidemicus* fermentation (the source of HA in FP-MD) began in 1997 [48,49] and has been studied in various ways to increase productivity [50,51]. The safety of HA has been demonstrated in multiple studies worldwide [52].

This 12-week intervention trial demonstrated that intake of FP-MD, a unique formulation of three synergistic ingredients (e.g., omega-3 fatty acids (EPA/DHA), astaxanthin, and lower molecular weight HA), can provide joint health benefits. The efficacy and safety profile in this study is consistent with the efficacy and safety reported in a previous study of FP-MD in a predominantly White population [29]. Although degenerative arthritis may have different specific causes and biological mechanisms, inflammation is a common feature of many forms of arthritis, and reduction in the pain caused by arthritis is an important part of arthritis treatment success [53].

Test product adherence and the occurrence of AEs are important indicators that can provide insight into the safety of raw materials in dietary supplements [54]. The rate of AEs in the FP-MD group was low and less than that of the placebo group. Overall, the high adherence rate, low AE rate, and lack of AE-related withdrawals in the FP-MD group indicate FP-MD is well tolerated.

As with all clinical trials, this study had several limitations. Although this study included only Korean participants, the results should be generalizable to other populations. The 12-week duration provided a reasonable time frame to assess FP-MD efficacy and safety; however, long-term studies are needed to demonstrate sustained effectiveness and provide additional safety data. Only participants with Kellgren and Lawrence Grade 1 or 2 osteoarthritis were evaluated; therefore, the effect of FP-MD on pain and physical function in individuals with moderate or severe degenerative arthritis is unknown.

This randomized controlled trial demonstrated statistically significant improvements in K-VAS pain scores and K-WOMAC total and subscale scores for participants taking FP-MD compared with placebo after 12 weeks of supplementation, confirming that this functional food can effectively address joint pain, the main symptom of degenerative arthritis, and improve physical function. Based on these clinical trial results and previously reported long-term safety data, the unique formulation of FP-MD, including krill oil, astaxanthin, and HA, is suggested as a dietary supplement to potentially reduce joint pain and improve physical function in individuals with mild osteoarthritis.

## Figures and Tables

**Table 1:** Composition of FP-MD and placebo capsules.

Composition	FP-MD 600 mg mg (%)	Placebo 600 mg mg (%)
FP-MD	462 (77)	0
Antarctic krill oil	321 (53.5)	0
*Haematococcus pluvialis* extract (to deliver 2 mg astaxanthin)	25–35 (4–5)	0
Sodium hyaluronate	33 (5.5)	0
Excipients	73–83 (12–14) *	
Palm oil	0	400 (67)
Olive oil	0	62 (10)
Soybean oil	114 (19)	114 (19)
Beeswax	24 (4)	24 (4)

* Amount (mg) depends on quantity of *Haematococcus pluvialis* extract.

**Table 2:** Improvement assessment score.

Score	Degree	Symptoms
1	Much better	Significant improvement in symptoms
2	Better	Overall improvement of symptoms
3	No change	No difference from baseline
4	Worse	Overall worsening of symptoms
5	Worsen	Significant worsening of symptoms

**Table 3:** Participant baseline demographic and lifestyle characteristics (per protocol set).

		FP-MD n = 37	Placebo n = 38	Total n = 75	*p*-Value
Sex n (%)	Male	16 (43.24)	15 (39.47)	31 (41.33)	0.7403 ^†^
	Female	21 (56.76)	23 (60.53)	44 (58.67)	
Age (y)	Mean ± SD	57.0 ± 10.28	59.0 ± 11.82	58.0 ± 11.06	0.2370 ^&^
	Min, Max	31.0, 70.0	35.0, 75.0	31.0, 75.0	
Exercise frequency (%)	None	7 (18.9)	6 (15.8)	13 (17.3)	0.9100 ^†^
	<3 sessions/week or <30 min/session	15 (40.5)	15 (39.5)	30 (40.00)	
	≥3 sessions/week or >30 min/session	15 (40.5)	17 (44.7)	32 (42.7)	
Smoking status n (%)	Non-smoker	31 (83.8)	32 (84.2)	63 (84.0)	0.1569 ^‡^
	Ex-smoker Stopped smoking > 6 months before screening visit	0	3 (7.9)	3 (4.0)	
	Smoker	6 (16.2)	3 (7.9)	9 (12.0)	
Cigarettes/day (among smokers)	Mean ± SD	11.7 ± 4.08	11.7 ± 7.6	11.7 ± 5.0	0.8774 ^&^
	Min, max	10.0, 20.0	5.0, 20.0	5.0, 20.0	
Smoking (years) (among smokers)	Mean ± SD	26.0 ± 9.38	21.7 ± 7.64	24.6 ± 8.62	0.5141 *
	Min, max	10.0, 36.0	15.0, 30.0	10.0, 36.0	
Alcohol consumption n (%)	None	22 (59.5)	20 (52.6)	42 (56.0)	0.5829 ^‡^
	Quit	1 (2.7)	1 (2.6)	2 (2.7)	
	<1 bottle/week	5 (13.5)	10 (26.3)	15 (20.0)	
	1~2 bottles/week	7 (18.9)	4 (10.5)	11 (14.7)	
	>3 bottles/weeks	2 (5.4)	3 (7.9)	5 (6.7)	
Body height (cm)	Mean ± SD	161.9 ± 10.24	160.8 ± 9.74	161.4 ± 9.94	0.6259 ^&^
	Min, max	145.2, 184.1	145.1, 180.0	145.1, 184.1	

* Compared between groups; *p*-value based on 2-sample *t*-test. ^&^ Compared between groups; *p*-value based on Wilcoxon rank–sum test. ^†^ Compared within groups; *p*-value based on Chi-square test. ^‡^ Compared within groups; *p*-value based on Fisher exact test.

**Table 4:** K-VAS scores (per protocol set).

K-VAS (mm)	FP-MD (n = 37)	Placebo (n = 38)	*p*-Value	*p*-Value ^‡^
	Mean ± SD	Mean ± SD		
Baseline	46.1 ± 9.77	42.7 ± 8.38	0.1596 ^†^	
Week 6	35.1 ± 17.13	34.6 ± 16.49	0.1059 ^†^	0.0854
Change from baseline	−11.0 ± 12.62	−8.1 ± 11.87		
*p*-value	<0.0001 **	<0.0001 ^#^		
Week 12	25.3 ± 16.39	32.1 ± 19.08	0.0105 *	0.0255
Change from baseline	−20.8 ± 16.16	−10.6 ± 17.58		
*p* value **	<0.0001	0.0007		

* Compared between groups; *p*-value based on 2-sample t-test. ^†^ Compared between groups; *p*-value based on Wilcoxon rank–sum test. ** Compared within groups; *p*-value based on paired *t*-test. ^#^ Compared within groups; *p*-value based on Wilcoxon signed–rank test. ^‡^ Compared between groups; *p*-value based on ANCOVA adjusted by baseline and adherence.

**Table 5:** K-WOMAC total and subscales scores (per protocol set).

	FP-MD (n = 38)	Placebo (n = 37)	*p*-Value	*p*-Value ^‡^
	Mean ± SD	Mean ± SD		
Total score				
Baseline	30.7 ± 14.81	28.3 ± 13.55	0.4737 *	
Week 6	21.2 ± 13.10	23.5 ± 13.75	0.1304 *	0.1658
Change from baseline	−9.5 ± 12.57	−4.8 ± 14.10		
*p*-value **	<0.0001	0.0432		
Week 12	17.7 ± 15.06	22.8 ± 15.07	0.0489 *	0.1063
Change from baseline	−13.0 ± 13.62	−5.5 ± 18.08		
*p*-value **	<0.0001	0.0674		
2. Pain score				
Baseline	6.0 ± 3.22	5.7 ± 2.64	0.6582 ^†^	
Week 6	4.0 ± 2.74	4.7 ± 2.98	0.1675 *	0.1149
Change from baseline	−2.0 ± 3.14	−1.0 ± 6.07		
*p*-value **	0.0004	0.0518		
Week 12	3.5 ± 2.99	4.5 ± 3.45	0.02635 ^†^	0.1779
Change from baseline	−2.5 ± 2.92	−1.3 ± 3.94		
*p*-value	<0.0001 **	0.0173 ^#^		
3. Stiffness score				
Baseline	2.9 ± 1.61	2.3 ± 1.44	0.5240 ^†^	
Week 6	2.0 ± 1.31	2.1 ± 1.35	0.4294 ^†^	0.0854
Change from baseline	−0.9 ± 1.78	−0.5 ± 1.45		
*p*-value	0.0040 ^#^	0.0310 **		
Week 12	1.8 ± 1.57	2.0 ± 1.62	0.2819 ^†^	0.0255
Change from baseline	−1.1 ± 2.08	−0.6 ± 1.79		
*p*-value	0.0039 **	0.0282 ^#^		
4. Physical function score				
Baseline	21.8 ± 11.03	20.0 ± 10.25	0.4639 *	
Week 6	15.2 ± 9.84	16.1 ± 10.14	0.1528 *	0.2148
Change from baseline	−6.6 ± 9.25	−3.3 ± 10.80		
*p*-value **	0.0001	0.0705		
Week 12	12.4 ± 10.83	16.3 ± 10.86	0.0398 *	0.0890
Change from baseline	−9.4 ± 9.99	−3.7 ± 13.38		
*p*-value **	<0.0001	0.1005		

* Compared between groups; *p*-value based on 2-sample *t*-test. ^†^ Compared between groups; *p*-value based on Wilcoxon rank–sum test. ** Compared within groups; *p*-value based on paired *t*-test. ^#^ Compared within groups; *p*-value based on Wilcoxon signed–rank test. ^‡^ Compared between groups; *p*-value based on ANCOVA adjusted by baseline factors and adherence.

**Table 6:** Investigator and participant improvement assessment scores (per protocol set).

	FP-MD (n = 37)	Placebo (n = 38)	*p*-Value ^†^
	Mean ± SD	Mean ± SD	
Investigator assessment score			
Week 6	2.4 ± 0.68	2.7 ± 0.53	0.0238
Week 12	2.1 ± 0.74	2.5 ± 0.72	0.0127
Participant assessment score			
Week 6	2.4 ± 0.65	2.7 ± 0.45	0.0194
Week 12	2.1 ± 0.70	2.6 ± 0.80	0.0070

^†^ Compared between groups; *p*-value based on Wilcoxon rank–sum test.

**Table 7:** Summary of adverse events (safety set).

	FP-MD (n = 50)			Placebo (n = 50)			*p*-Value
	n	Incidence (%)	Cases	n	Incidence (%)	Cases	
Adverse events (AEs)	2	4.0	2	8	16.0	10	0.0455 ^†^
Serious AEs (SAE)	1	2.0	1	2	4.0	2	1.0000 ^‡^
Dropouts due to AEs	0	0.0	0	1	2.0	1	1.0000 ^‡^

^†^ *p*-value based on Chi-square test. ^‡^ *p*-value based on Fisher exact test.

## Data Availability

Data described in this study will be made available upon request pending application and approval from the corresponding author.

## Supplementary Materials

The following supporting information can be downloaded at: https://www.mdpi.com/article/10.3390/nu15173769/s1, Table S1: Hematologic Tests (Safety Set); Table S2: Blood Chemistry and Lipid Tests (Safety Set); Table S3: Vital Signs and Body Weight (Safety Set); Table S4: ECG Evaluation (Safety Set).
