# Correction: Hill et al. A Multicenter, Randomized, Double-Blinded, Placebo-Controlled Clinical Trial to Evaluate the Efficacy and Safety of a Krill Oil, Astaxanthin, and Oral Hyaluronic Acid Complex on Joint Health in People with Mild Osteoarthritis. *Nutrients* 2023, *15*, 3769

**DOI:** 10.3390/nu16121961

**Published:** 2024-06-20

**Authors:** W. Stephen Hill, Margaret H. Dohnalek, Yejin Ha, Seok-Jung Kim, Jae-Chul Jung, Seung-Baik Kang

**Affiliations:** 1 US Nutraceuticals, Inc. d/b/a Valensa International, Eustis, FL 32726, USA; s.hill@valensa.com (W.S.H.); m.dohnalek@valensa.com (M.H.D.); 2 NOVAREX Co., Ltd., 80, Osongsaengmyeong 14-ro, Osong-eup, Chueongju-si 28220, Republic of Korea; yj9113@novarex.co.kr; 3 Department of Orthopedic Surgery, Uijeongbu St. Mary’s Hospital, College of Medicine, The Catholic University of Korea, Cheonbo-ro, Uijeongbu-si 11765, Republic of Korea; peter@catholic.ac.kr; 4 Department of Orthopedic Surgery, Seoul National University College of Medicine, Boramae Hospital, Seoul 07061, Republic of Korea

## 1. Explanation and Summary of Corrections

In the original publication [1], there was an error in reporting the results of the statistical analysis for the Korean Western Ontario and McMaster Universities Osteoarthritis Index (K-WOMAC) scores for Week 12. In the statistical analysis, there was no statistically significant difference in K-WOMAC pain scores between the FlexPro MD^®^ (FP-MD) and placebo groups at Week 12, as reported in the original manuscript. However, there was a statistically significant difference between the study groups for the K-WOMAC physical function scores at this timepoint. Therefore, corrections were required in the following sections of the manuscript: Abstract, Results 3.3 K-WOMAC (e.g., text, Table 5, and Figure 3), and Discussion. Each correction is detailed below.

## 2. Error in Abstract

In the original publication [1], there was an error in reporting the K-WOMAC score results in the Abstract. The sentence in the original Abstract stated “The Korean Western Ontario and McMaster Universities Osteoarthritis Index (K-WOMAC) total score was also significantly improved in the FP-MD group at week 12 compared with placebo (−13.0 ± 13.62 vs. −5.5 ± 18.08, *p* = 0.0489), especially an improvement in pain score (–2.5 ± 2.92 vs. −1.3 ± 3.94, *p* = 0.02635)”.

The corrected sentence states “The Korean Western Ontario and McMaster Universities Osteoarthritis Index (K-WOMAC) total score was also significantly improved in the FP-MD group at week 12 compared with placebo (−13.0 ± 13.62 vs. −5.5 ± 18.08, *p* = 0.0489), especially an improvement in the physical function score (−9.4 ± 9.99 vs. −3.7 ± 13.38, *p* = 0.0398)”.

## 3. Error in Results

In the original publication [1], there was an error in reporting the mean changes from baseline to week 12 in the K-WOMAC subscale scores in the second paragraph of Section 3.3 K-WOMAC. The sentence in the original manuscript stated “However, at week 12, pain, stiffness, and physical function subscale scores were significantly lower in participants taking FP-MD (Table 5; Figure 3)”.

The corrected sentence states “However, at week 12, the physical function score was significantly lower in participants taking FP-MD *(p* = 0.0398)”.

Table 5 also required corrections. For the week 12 pain score, the *p*-value for the between-groups unadjusted analysis of the change from baseline was corrected from 0.02635 to 0.2635.

The baseline stiffness score for the placebo group was corrected from 2.3 ± 1.44 to 2.6 ± 1.44.

For the week 6 stiffness score, the *p*-value for the between-groups adjusted analysis of the change from baseline was corrected from 0.0854 to 0.5298. For the week 12 stiffness score, the *p*-value for the between-groups adjusted analysis of the change from baseline was corrected from 0.0255 to 0.6330.

For the physical function score, the week 6 baseline value for the placebo group was adjusted from 16.1 to 16.7.

Corrected Table 5 appears below.

**Table 5:** K-WOMAC total and subscales scores (per protocol set).

	FP-MD (n = 38)	Placebo (n = 37)	*p*-Value	*p*-Value ^‡^
	Mean ± SD	Mean ± SD		
1. Total score				
Baseline	30.7 ± 14.81	28.3 ± 13.55	0.4737 *	
Week 6	21.2 ± 13.10	23.5 ± 13.75	0.1304 *	0.1658
Change from baseline	−9.5 ± 12.57	−4.8 ± 14.10		
*p*-value **	<0.0001	0.0432		
Week 12	17.7 ± 15.06	22.8 ± 15.07	0.0489 *	0.1063
Change from baseline	−13.0 ± 13.62	−5.5 ± 18.08		
*p*-value **	<0.0001	0.0674		
2. Pain score				
Baseline	6.0 ± 3.22	5.7 ± 2.64	0.6582 ^†^	
Week 6	4.0 ± 2.74	4.7 ± 2.98	0.1675 *	0.1149
Change from baseline	−2.0 ± 3.14	−1.0 ± 6.07		
*p*-value **	0.0004	0.0518		
Week 12	3.5 ± 2.99	4.5 ± 3.45	0.2635 ^†^	0.1779
Change from baseline	−2.5 ± 2.92	−1.3 ± 3.94		
*p*-value	<0.0001 **	0.0173 ^#^		
3. Stiffness score				
Baseline	2.9 ± 1.61	2.6± 1.44	0.5240 ^†^	
Week 6	2.0 ± 1.31	2.1 ± 1.35	0.4294 ^†^	0.5298
Change from baseline	−0.9 ± 1.78	−0.5 ± 1.45		
*p*-value	0.0040 ^#^	0.0310 **		
Week 12	1.8 ± 1.57	2.0 ± 1.62	0.2819 ^†^	0.6330
Change from baseline	−1.1 ± 2.08	−0.6 ± 1.79		
*p*-value	0.0039 **	0.0282 ^#^		
4. Physical function score				
Baseline	21.8 ± 11.03	20.0 ± 10.25	0.4639 *	
Week 6	15.2 ± 9.84	16.7 ± 10.14	0.1528 *	0.2148
Change from baseline	−6.6 ± 9.25	−3.3 ± 10.80		
*p*-value **	0.0001	0.0705		
Week 12	12.4 ± 10.83	16.3 ± 10.86	0.0398 *	0.0890
Change from baseline	−9.4 ± 9.99	−3.7 ± 13.38		
*p*-value **	<0.0001	0.1005		

* Compared between groups; *p*-value based on 2-sample *t*-test. ^†^ Compared between groups; *p*-value based on Wilcoxon rank-sum test. ** Compared within groups; *p*-value based on paired *t*-test. ^#^ Compared within groups; *p*-value based on Wilcoxon signed–rank test. ^‡^ Compared between groups; *p*-value based on ANCOVA adjusted by baseline factors and adherence.

Figure 3 also required corrections. The *p*-value for the K-WOMAC pain score was deleted, and the *p*-value for the K-WOMAC physical function score was added. A typographical error in the x-axis label “Physical function” was also corrected.

Corrected Figure 3 appears below.

**Figure 3:**
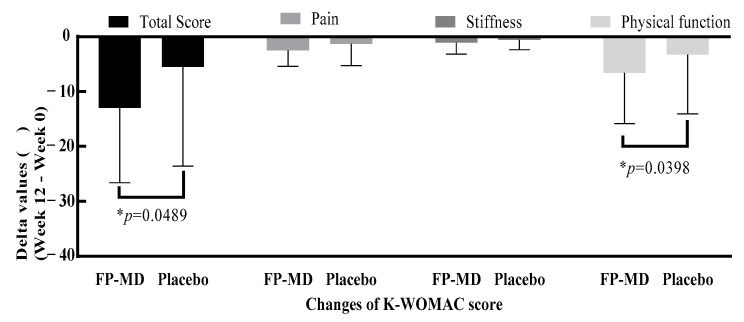
Changes in K-WOMAC score after 12 weeks of FP-MD intake compared with placebo. FP-MD, FlexPro MD^®^; K-WOMAC, Korean Western Ontario and McMaster Universities Osteoarthritis Index. * *p*-value based on 2-sample *t*-test.

## 4. Error in Discussion

In the original publication [1], the first sentence of the last paragraph of the Discussion section stated “This randomized controlled trial demonstrated statistically significant improvements in K-VAS pain scores and K-WOMAC total and subscale scores for participants taking FP-MD compared with placebo after 12 weeks of supplementation, confirming that this functional food can effectively address joint pain, the main symptom of degenerative arthritis, and improve physical function”.

The corrected sentence states “This randomized controlled trial demonstrated statistically significant improvements in K-VAS pain scores and K-WOMAC total and subscale scores for physical function for participants taking FP-MD compared with placebo after 12 weeks of supplementation, confirming that this functional food can effectively address degenerative arthritis and improve physical function”.

The authors apologize for these mistakes in presenting the statistical analysis of the study data. The overall benefits of supplementation with FP-MD compared with the placebo, as determined in this clinical trial, include total joint health and a statistically significant improvement in physical function. All other scientific conclusions of the original publication are unaffected. This correction was approved by the Academic Editor. The original publication was also updated.
