# Supplementary material for: A Multicenter, Randomized, Double-Blinded, Placebo-Controlled Clinical Trial to Evaluate the Efficacy and Safety of a Krill Oil, Astaxanthin, and Oral Hyaluronic Acid Complex on Joint Health in People with Mild Osteoarthritis
Source: Nutrients. 2023 Aug 29;15(17):3769. doi: 10.3390/nu15173769 (PMC10490060; doi:10.3390/nu15173769)
Supplement: Supplementary file 1 [file nutrients-15-03769-s001.zip › nutrients-2548786-supplementary.pdf]

A Multicenter, Randomized, Double-Blinded, Placebo-Controlled Clinical Trial to Evaluate the Efficacy and Safety of a Krill Oil, Astaxanthin, and Oral Hyaluronic Acid Complex on Joint Health in People with Mild Osteoarthritis

W. Stephen Hill, Margaret H. Dohnalek, YeJin Ha, Seok Jung Kim, Jae-Chul Jung, and Seung-Baik Kang

Supplementary Tables S1–S4

Table S1. Hematologic Tests (Safety Set)

		FP-MD n=50		Placebo n=50		p-value
		n	Mean±SD	n	Mean±SD	
WBC (10³/μL)	Baseline	50	5.77±1.35	50	5.56±1.40	0.2159&
	Week 12	48	5.85±1.43	45	5.68±1.28	
	Change from baseline	48	0.08±0.87	45	0.05±1.21	0.8813*
	p-value**		0.5199		0.7900	
RBC (10⁶/μL)	Baseline	50	4.54±0.39	50	4.59±0.48	0.9533&
	Week 12	48	4.49±0.40	45	4.51±0.46	
	Change from baseline	48	-0.04±0.21	45	-0.07±0.18	0.4987*
	p-value**		0.1741		0.0117	
Hb (g/dL)	Baseline	50	14.12±1.35	50	14.21±1.48	0.9395&
	Week 12	48	13.94±1.39	45	13.97±1.36	
	Change from baseline	48	-0.16±0.66	45	-0.28±0.54	0.3400*
	p-value**		0.0964		0.0011	
Hct (%)	Baseline	50	41.99±3.32	50	42.13±3.77	0.9807&
	Week 12	48	41.50±3.33	45	41.39±3.62	
	Change from baseline	48	-0.43±1.74	45	-0.80±1.59	0.2838*
	p-value**		0.0941		0.0015	
Platelet count (10³/μL)	Baseline	50	239.88±44.96	50	227.94±43.18	0.1787*
	Week 12	48	238.15±50.40	45	221.67±47.08	
	Change from baseline	48	-0.81±22.03	45	-4.93±22.74	0.3771*
	p-value**		0.7994		0.1526	
Neutrophil (%)	Baseline	50	52.86±9.32	50	50.72±9.06	0.2474*
	Week 12	48	51.11±8.13	45	50.86±8.55	
	Change from baseline	48	-1.81±7.70	45	0.42±8.04	0.6391&
	p-value		0.3410#		0.7304**	
Lymphocyte (%)	Baseline	50	36.99±8.97	50	39.16±8.16	0.2104*
	Week 12	48	38.76±7.63	45	39.12±7.40	
	Change from baseline	48	1.72±6.64	45	-0.29±7.22	0.5284&
	p-value		0.2747#		0.7881**	
Monocyte (%)	Baseline	50	7.05±1.89	50	7.09±1.61	0.9176&
	Week 12	48	7.06±1.62	45	7.12±1.58	
	Change from baseline	48	0.00±1.77	45	0.00±1.46	0.9571&

	p-value		0.7811#		0.9919**	
Eosinophil (%)	Baseline	50	2.55±1.86	50	2.48±1.92	0.9642&
	Week 12	48	2.51±2.13	45	2.35±1.97	
	Change from baseline	48	0.07±1.39	45	-0.12±1.34	0.8027&
	p-value#		0.8047		0.4684	
Basophil (%)	Baseline	50	0.55±0.26	50	0.56±0.33	0.8078&
	Week 12	48	0.56±0.25	45	0.55±0.32	
	Change from baseline	48	0.02±0.22	45	-0.01±0.26	0.5462*
	p-value**		0.5541		0.7731	

*Compared between groups; p-value based on 2-sample *t*-test.
&Compared between groups; p-value based on Wilcoxon rank–sum test.
**Compared within groups; p-value based on paired *t*-test.
#Compared within groups; p-value based on Wilcoxon signed–rank test.

Table S2. Blood Chemistry and Lipid Tests (Safety Set)

		n	FP-MD N=50 Mean±SD	n	Placebo N=50 Mean±SD	p-value
Glucose (mg/dL)	Baseline	50	103.32±22.82	50	105.94±17.10	0.2564&
	Week 12	48	102.44±14.56	45	107.76±19.57	
	Change from baseline	48	-1.00±15.28	45	0.87±13.03	0.6777&
	p-value#		0.7694		0.8167	
AST(GOT) (IU/L)	Baseline	50	24.72±7.63	50	25.88±12.44	0.8845&
	Week 12	48	23.17±5.76	45	25.07±11.03	
	Change from baseline	48	-1.75±5.74	45	-0.82±6.10	0.2766&
	p-value#		0.0187		0.4404	
ALT(GPT) (IU/L)	Baseline	50	23.92±10.08	50	25.40±15.51	0.5164&
	Week 12	48	22.69±9.42	45	24.47±15.11	
	Change from baseline	48	-1.58±7.61	45	-1.38±11.24	0.5224&
	p-value#		0.0280		0.4404	
Creatinine (mg/dL)	Baseline	50	0.82±0.22	50	0.83±0.22	0.9161*
	Week 12	48	0.84±0.21	45	0.83±0.21	
	Change from baseline	48	0.02±0.09	45	-0.01±0.08	0.1076*
	p-value**		0.1473		0.4315	
Triglyceride (mg/dL)	Baseline	50	128.56±105.57	50	124.60±73.36	0.9368&
	Week 12	48	126.25±59.17	45	126.02±78.31	
	Change from baseline	48	-2.67±103.34	45	1.62±66.09	0.2304&
	p-value#		0.3225		0.5370	
Total Cholesterol (mg/dL)	Baseline	50	199.10±42.85	50	192.22±29.26	0.3511*
	Week 12	48	190.69±38.38	45	188.07±26.92	
	Change from baseline	48	-9.60±30.08	45	-6.64±20.79	0.7321&
	p-value		0.1211#		0.0376**	
HDL-Cholesterol (mg/dL)	Baseline	50	57.84±13.95	50	57.70±13.52	0.7747&
	Week 12	48	56.81±11.42	45	55.78±12.75	
	Change from baseline	48	-1.56±8.57	45	-1.98±7.51	0.4460&
	p-value		0.3806#		0.0843**	
LDL-Cholesterol (mg/dL)	Baseline	50	116.50±37.26	50	110.62±27.32	0.3983&
	Week 12	48	111.44±35.61	45	109.91±27.64	
	Change from baseline	48	-5.69±29.89	45	-2.89±17.98	0.7760&
	p-value		0.5536#		0.2870**	
Uric acid (mg/dL)	Baseline	50	5.05±1.19	50	5.27±1.59	0.7906&
	Week 12	48	4.95±1.19	45	5.42±1.77	
	Change from baseline	48	-0.13±0.77	45	0.16±0.68	0.0646*
	p-value**		0.2612		0.1295	

*Compared between groups; p-value based on 2-sample *t*-test.
&Compared between groups; p-value based on Wilcoxon rank–sum test.
**Compared within groups; p-value based on paired *t*-test.
#Compared within groups; p-value based on Wilcoxon signed–rank test.

Table S3. Vital Signs and Body Weight (Safety Set)

		FP-MD N=50		Placebo N=50		p-value
		n	Mean±SD	n	Mean±SD	
Pulse (bpm)	Baseline	50	72.44±10.23	50	74.50±9.67	0.2448&
	Week 6	49	72.92±9.07	46	75.48±7.99	
	Change from baseline	49	0.88±6.88	46	0.87±8.72	0.9960*
	p-value**		0.3767		0.5023	
	Week 12	48	70.52±8.37	45	72.93±7.87	
	Change from baseline	48	-1.77±6.32	45	-1.96±7.54	0.8981*
	p-value**		0.0581		0.0888	
Systolic blood pressure (mm Hg)	Baseline	50	128.52±11.71	50	129.28±9.09	0.8091&
	Week 6	49	129.67±10.78	46	128.70±10.18	
	Change from baseline	49	1.06±12.33	46	-0.78±8.74	0.4007*
	p-value**		0.5498		0.5468	
	Week 12	48	126.92±12.44	45	127.47±8.79	
	Change from baseline	48	-1.75±10.75	45	-2.47±9.18	0.7311*
	p-value**		0.2650		0.0783	
Diastolic blood pressure (mm Hg)	Baseline	50	82.36±8.93	50	82.58±8.48	0.8997*
	Week 6	49	82.10±7.66	46	81.41±8.85	
	Change from baseline	49	-0.61±7.11	46	-1.33±8.54	0.6582*
	p-value**		0.5494		0.2981	
	Week 12	48	80.88±8.63	45	81.38±8.12	
	Change from baseline	48	-1.88±6.83	45	-1.76±7.37	0.9355*
	p-value**		0.0635		0.1171	
Weight (kg)	Baseline	50	65.02±11.02	50	64.70±13.72	0.5192&
	Week 6	49	65.26±11.19	46	64.75±13.60	
	Change from baseline	49	0.32±1.28	46	0.19±0.79	0.8639&
	p-value		0.1588#		0.1123**	
	Week 12	48	64.25±11.34	45	64.48±13.69	
	Change from baseline	48	-0.29±2.07	45	-0.43±2.36	0.3101&
	p-value#		0.0578		0.5362	

*Compared between groups; p-value based on 2-sample *t*-test.
&Compared between groups; p-value based on Wilcoxon rank–sum test.
**Compared within groups; p-value based on paired *t*-test.
#Compared within groups; p-value based on Wilcoxon signed–rank test.

Table S4. ECG Evaluation (Safety Set)

		Week 12	
	**Baseline**	**Normal n (%)**	**Abnormal n (%)**
FP-MD (n=48)	Normal	48 (100)	0 (0)
	Abnormal	0 (0)	0 (0)
Placebo (n=45)	Normal	45 (100)	0 (0)
	Abnormal	0 (0)	0 (0)
